# Mechanistic Insights into Cooling-Rate-Governed Acicular Ferrite Transformation Kinetics and Strengthening-Toughening Synergy in EH36 Heavy Steel Plate

**DOI:** 10.3390/ma18204661

**Published:** 2025-10-10

**Authors:** Chunliang Yan, Fengming Wang, Rongli Sang, Qingjun Zhang

**Affiliations:** 1 Comprehensive Testing and Analyzing Center, North China University of Science and Technology, Tangshan 063210, China; yanchunliang@ncst.edu.cn (C.Y.); sangrongli@ncst.edu.cn (R.S.); 2 College of Metallurgy and Energy, North China University of Science and Technology, Tangshan 063210, China; 3 College of Science, North China University of Science and Technology, Tangshan 063210, China; wfm77@ncst.edu.cn

**Keywords:** keyword acicular ferrite, cooling rate, oxide metallurgy, high-temperature in situ observation, kinetics

## Abstract

This study was focused on addressing the performance degradation in core microstructures of ultra-heavy steel plates (thickness ≥ 50 mm) caused by non-uniform cooling during thermo-mechanical controlled processing. Using microalloyed DH36 steel as the research subject, we systematically investigated the effects of cooling rate on the nucleation and growth of acicular ferrite and its consequent microstructure-property relationships through an integrated approach combining in situ observation via high-temperature laser scanning confocal microscopy with multiscale characterization techniques. Results demonstrate that the cooling rate significantly affects acicular ferrite formation, with the range of 3–7 °C/s being most conducive to acicular ferrite formation. At 5 °C/s, the acicular ferrite volume fraction reached a maximum of 74% with an optimal aspect ratio (5.97). Characterization confirmed that TiOx-Al_2_O_3_·SiO_2_-MnO-MnS complex inclusions act as effective nucleation sites for acicular ferrite, where the MnS outer layer plays a key role in reducing interfacial energy and promoting acicular ferrite radial growth. Furthermore, the interlocking acicular ferrite structure was shown to enhance microhardness by 14% (HV0.1 = 212.5) compared to conventional ferrite through grain refinement strengthening and dislocation strengthening (with a dislocation density of 2 × 10^8^ dislocations/mm^2^). These results provide crucial theoretical insights and a practical processing window for strengthening-toughening control of heavy plate core microstructures, offering a viable pathway for improving the comprehensive performance of ultra-heavy plates.

## 1. Introduction

Medium and heavy steel plates serve as critical structural materials and play an irreplaceable role in energy equipment, marine engineering, and other fields. With the development of large-scale engineering equipment, there is an increasing demand for enhanced strength-toughness matching in extra-heavy plates (thickness ≥ 50 mm). Although traditional hot-working processes can effectively improve the properties of high-strength low-alloy steels [1,2,3,4,5], significant challenges remain in their application to thick plates: uneven cooling through the thickness direction leads to a section effect (with the cooling rate at the core being only 10–30% of that at the surface), resulting in microstructural gradients and inhomogeneous mechanical properties in heavy steel plates. The internal quality of such plates often fails to meet required standards [6,7]. Existing studies have primarily focused on mitigating these defects through compositional adjustments or process optimizations [8,9], yet a systematic understanding of the quantitative relationships among cooling rate, phase transformation behavior, and mechanical properties is still lacking. In particular, there is a scarcity of in situ experimental evidence on dynamic phase transformations in thick plates.

In recent years, acicular ferrite (AF) has emerged as a potential solution for improving the core properties of thick plates due to its interlocking microstructure and grain refinement strengthening effect. The mechanism of inclusion-induced AF nucleation has been extensively studied in weld heat-affected zones (HAZ) [10,11,12,13,14,15,16]. Numerous studies have investigated the nucleation and growth of AF and its beneficial effects on macroscopic properties [17,18,19,20,21,22,23,24,25,26], revealing that in addition to finely dispersed effective oxides, heat treatment processes and cooling rates also significantly influence AF formation. For instance, Liu et al. [27] found that Mo-containing low-carbon steels can form AF structures even at relatively low cooling rates. Zhu et al. [28] demonstrated through oxide metallurgy-based microalloying research that AF can be obtained within a cooling rate range of 0.3–50 °C/s, with low-carbon steels particularly prone to forming AF at cooling rates of 10–30 °C/s. Liu et al. [29], in their study on 420 MPa grade ultra-high-strength marine steels, observed that only AF forms when the cooling rate exceeds 7 °C/s, and it remains stable across a considerable cooling range. Shim [30] obtained a microstructure rich in AF in low-carbon steel at a cooling rate of 5 °C/s. Wang et al. [31] reported that the growth rate of AF increases with higher cooling rates: at cooling rates of 2, 5, and 15 °C/s, the average growth rates of AF were 2.2, 6.6, and 14.4 μm/s, respectively. Yang et al. [32] investigated ferrite transformation behavior under different cooling rates and found that at 10 °C/s, the transformation start temperatures for sideplate ferrite and AF decreased to 560 °C from initial values of 700 °C and 650 °C, respectively. This suggests a correlation between the proportion of AF and the transformation temperature.

Despite the extensive literature on the effects of cooling rate on low-carbon steel microstructures [33], quantitative investigations into the relationship between the cooling rate and acicular ferrite (AF) nucleation during heavy plate rolling remain relatively limited. The underlying mechanism through which cooling rate governs AF formation particularly requires in-depth exploration.

In this study, innovative in situ observation via high-temperature laser confocal microscopy is integrated with multi-scale characterization techniques. This study achieved direct visualization of the dynamic phase transformation of AF in EH36 heavy plate steel under varying cooling rates, thereby overcoming the limitations of conventional static analysis. Furthermore, we established quantitative correlations among cooling rate, transformation kinetics, microstructure, and mechanical properties, revealing the thermodynamic and kinetic mechanisms governing AF nucleation and growth. This work provides a novel theoretical foundation and precise processing window for strengthening-toughening control of the core microstructure in heavy steel plates, showing potential to advance plate steel production from empirical optimization toward precision design.

## 2. Materials and Methods

### 2.1. Experimental Materials

EH36 steel is a typical high-performance low-alloy steel widely used in marine engineering and other fields. It has attracted significant attention due to its high strength, good toughness, and excellent weldability [34,35]. In this study, based on EH36 steel, microalloying elements were introduced via oxide metallurgy technology to transform the final microstructure into an acicular ferrite (AF) structure, thereby significantly enhancing its toughness and strength while optimizing its weldability.

The experimental steel was smelted in a lab-made vacuum induction furnace at our institution. With the target composition of EH36 steel, a microalloying design was implemented based on the oxide metallurgy process. The primary raw material was industrial pure iron, and the alloying elements included aluminum, manganese, silicon, titanium, niobium, and vanadium. Specifically, Ti-Nb-V microalloying was conducted during the smelting process based on the oxide metallurgy approach to form effective inclusions for acicular ferrite nucleation. The chemical composition of the sample was determined using spark optical emission spectrometry, and the results are presented in Table 1. The composition met the target requirements. Heat treatment experiments were conducted on this experimental steel to analyze the growth kinetics mechanism of ferrite.

### 2.2. Experimental Methods

High-temperature in situ observation is an effective method for investigating the phase transformation processes in steels. Utilizing a high-temperature laser scanning confocal microscope (LSCM, model VL2000DX-SVF17SP, Lasertec Corporation, Yokohama, Japan), which offers precise temperature control and in situ observation capabilities. The system enables rapid temperature control (with an accuracy of ±0.1 °C) through an infrared light-collecting imaging heating mechanism, combined with a 405 nm laser light source, allowing real-time microscopic observation under an argon gas protective atmosphere. This technique enables the real-time recording of the nucleation and growth behaviors of intragranular ferrite, thereby revealing the influence of cooling rate on its precipitation kinetics.

**Figure 1:**
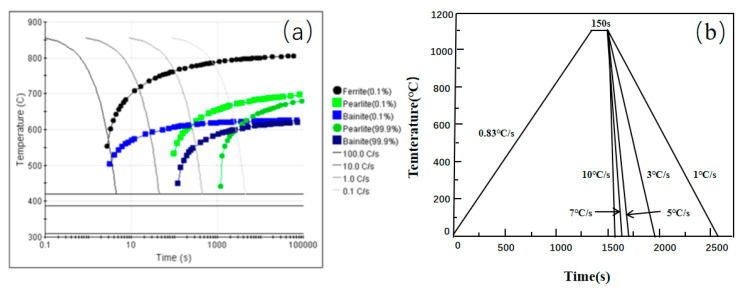
(**a**) Calculated continuous cooling transformation (CCT) diagram of the experimental steel; (**b**) Schematic of the applied heat treatment schedule.

The specific experimental procedure was as follows: Cylindrical specimens with dimensions of φ7 mm × 3 mm were machined from the ingot. After grinding and polishing, they were subjected to heat treatment experiments on the LSCM stage. The austenitization temperature was determined based on the steel’s composition using an empirical formula, yielding an Ac_3_ temperature of 843 °C. To ensure complete austenitization and carbide dissolution, the austenitization temperature for the experimental steel was set to 1100 °C. Furthermore, the Continuous Cooling Transformation (CCT) diagram for this steel composition was calculated using JMatPro software (version 8.0, Sente Software Ltd., Guildford, Surrey, UK), as shown in Figure 1a. Based on these foundations, the thermal schedule was designed as follows: the specimen was heated to 1100 °C at a rate of 0.83 °C/s, held for 150 s, and then cooled to room temperature at various rates of 1, 3, 5, 7, and 10 °C/s (Figure 1b).

### 2.3. Microstructural Characterization

The samples cooled to room temperature were prepared by polishing and etching to observe the microstructural evolution under different cooling rates.

(1) Metallographic observation: The specimens were ground, polished, and then etched with a 4% nital solution. Microstructural observation and analysis were conducted using a Leica DM2700M optical microscope (OM). The volume fraction of primary ferrite was statistically quantified.

(2) Scanning electron microscopy (SEM) analysis: A field-emission scanning electron microscope (FEI Scios) was employed to examine the morphology of inclusions and intragranular ferrite. The microscope was equipped with an energy dispersive spectrometer (EDS) for analyzing elemental distribution and composition to identify the types of inclusions that induce ferrite nucleation. Additionally, electron backscatter diffraction (EBSD) was utilized to characterize grain boundaries, phase distribution, as well as the grain orientation and misorientation distribution of ferrite.

(3) Transmission electron microscopy (TEM) analysis: A transmission electron microscope (JEM-2800) was used to observe the sub-micron morphology and crystal defects of inclusions, primary ferrite, and secondary ferrite. The attached EDS system was employed for compositional analysis of the inclusions.

### 2.4. Microhardness Measurement

The microhardness of the samples was measured using a Vickers microhardness tester (Falcon 501). Indentations were performed on different regions based on the microstructure revealed by etching to obtain representative hardness values.

To mitigate the limitations arising from the restricted observation area of small-scale samples and to ensure the reliability of microstructural statistical characterization, the following measures were implemented in this study to enhance the statistical significance of the results: at least three independent repeat experiments were conducted for each cooling condition, and microstructural observations and microhardness tests were performed on multiple areas (e.g., center, edge) of individual samples. The data reported herein are representative results obtained from this extensive set of measurements.

## 3. Results

### 3.1. Dynamic Observation of Acicular Ferrite Nucleation and Growth

**Figure 2:**
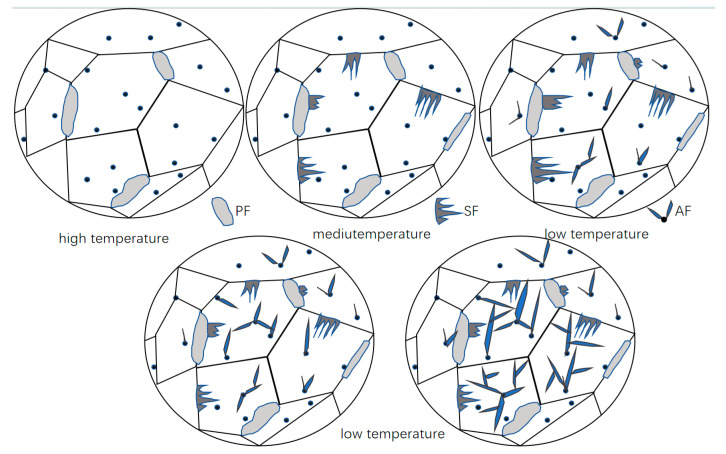
Schematic illustration of the sequential formation of polygonal ferrite (PF), sideplate ferrite (SF), and acicular ferrite (AF) during continuous cooling.

The microstructural analysis of samples subjected to different cooling rates reveals a common evolutionary pattern: the phase transformation follows a specific temperature sequence, wherein the morphology of the products and their formation order are governed by the competitive mechanism between the phase transformation driving force and carbon diffusion capacity. As shown in Figure 2, this schematic illustrates the typical formation sequence, nucleation sites, and morphological characteristics of polygonal ferrite (PF), sideplate ferrite (SF), and acicular ferrite (AF) during continuous cooling. Polygonal ferrite (PF) forms first in the high-temperature regime, followed by the precipitation of the sideplate ferrite (SF) in the medium-temperature regime, and finally, the transformation is completed by acicular ferrite (AF) in the low-temperature regime.

**Figure 3:**
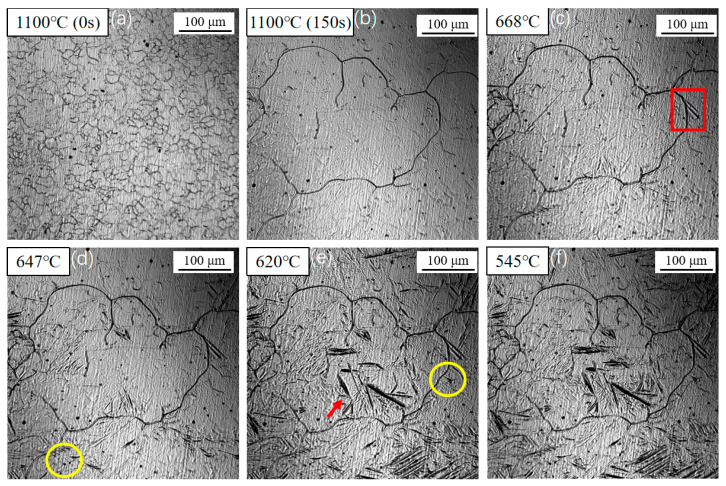
In situ observation of the nucleation and growth of acicular ferrite (AF) during cooling at 1 °C/s. (**a**–**c**) Nucleation of sideplate ferrite (SF) at austenite grain boundaries, region marked by the red box. (**d**) Nucleation of primary AF on an inclusion, region marked by the yellow circle. (**e**) Induced nucleation of secondary ferrite, (region marked by the red arrows) and coarsening of AF/SF. (**f**) Final microstructure after transformation completion.

Figure 3 shows the nucleation and growth of acicular ferrite (AF) during in situ cooling at 1 °C/s. After heating to 1100 °C and holding for 150 s, the prior austenite grains grew to approximately 100 μm. As the temperature decreased, sideplate ferrite (SF) began to nucleate at austenite grain boundaries around 668 °C and grew into the grain interiors (as indicated by the rectangle in Figure 3c). Upon further cooling to 647 °C, primary acicular ferrite (AF) started to nucleate on the surfaces of inclusions (as highlighted by the circle in Figure 3d). Concurrently, the length of the sideplate ferrite increased noticeably compared to that in Figure 3c. When the temperature reached 620 °C (Figure 3e), secondary ferrite was induced to nucleate on the broad faces of both the sideplate ferrite and the primary acicular ferrite (red arrows). Both the AF and SF coarsened due to carbon diffusion, resulting in a decrease in their aspect ratios. The transformation concluded at approximately 545 °C, as no further microstructural changes were observed within the field of view.

At relatively high temperatures, although the transformation driving force is modest, the strong carbon diffusion capacity dominates. Ferrite nuclei tend to form at the most energetically favorable austenite grain boundaries, leading to the preferential nucleation and slow growth of PF into equiaxed grains. As the temperature decreases, the transformation driving force increases, while the carbon diffusion capacity weakens. To achieve faster growth rates, the system favors ferrite growing parallelly from the grain boundaries into the austenite interior, forming SF with its characteristic parallel plate-like morphology. When the temperature further drops into the low-temperature regime, the significantly increased transformation driving force is sufficient to overcome the energy barrier for intragranular nucleation, promoting AF nucleation on inclusions. During the initial nucleation stage, multiple acicular ferrite (AF) laths typically nucleate on a single inclusion and commence growth within a short timeframe. Concurrently, the very limited carbon diffusion capacity restricts the coarsening of the ferrite plates. The newly formed AF laths exhibit a high aspect ratio and a distinct two-dimensional acicular morphology, bounded by well-defined ferrite/austenite interfaces. These individual AF laths undergo hard impingement, forming an interlocking microstructure that partitions the original austenite grain into isolated, smaller regions. Subsequent phase transformation is confined within these micro-domains. As the transformation proceeds, the available volume decreases and carbon becomes enriched in the remaining austenite, ultimately causing the cessation of AF lath growth. This interlocked and crossing AF structure effectively refines the final grain size.

### 3.2. Influence of Cooling Rate on the Transformation Kinetics of Acicular Ferrite

**Figure 4:**
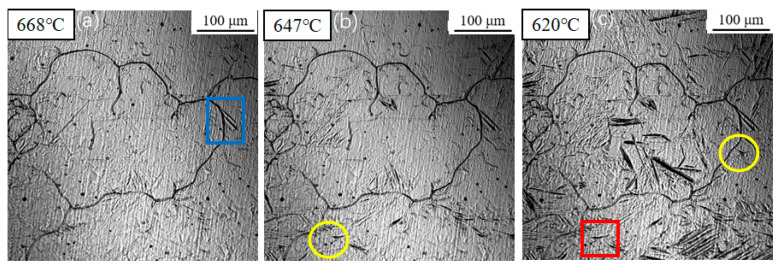
In situ observation of ferrite transformation at 1 °C/s: (**a**) T = 668 °C, growth of sideplate ferrite (SF), region marked by the blue box; (**b**) T = 647 °C, nucleation of acicular ferrite (AF) on an inclusion, region marked by the yellow circle; (**c**) T = 620 °C, formation of secondary ferrite, region marked by the red box.

**Figure 5:**
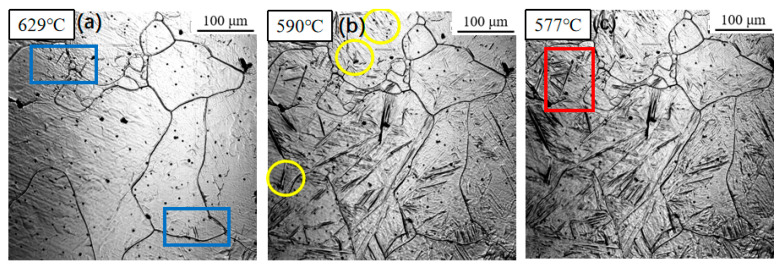
In situ observation of ferrite transformation at 3 °C/s: (**a**) T = 629 °C, growth of sideplate ferrite (SF), region marked by the blue box; (**b**) T = 590 °C, nucleation of acicular ferrite (AF) on an inclusion, region marked by the yellow circle; (**c**) T = 577 °C, formation of secondary ferrite, region marked by the red box.

**Figure 6:**
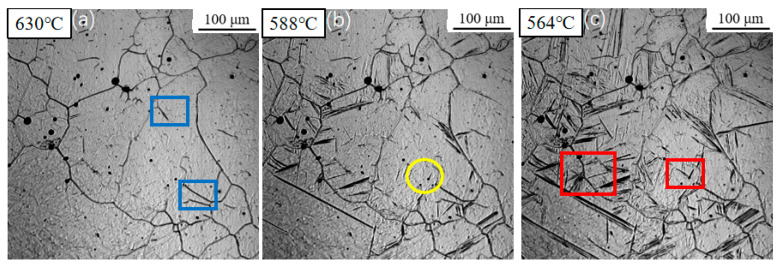
In situ observation of ferrite transformation at 5 °C/s: (**a**) T = 630 °C, growth of sideplate ferrite (SF), region marked by the blue box; (**b**) T = 588 °C, nucleation of acicular ferrite (AF) on an inclusion, region marked by the yellow circle; (**c**) T = 564 °C, formation of secondary ferrite, region marked by the red box.

**Figure 7:**
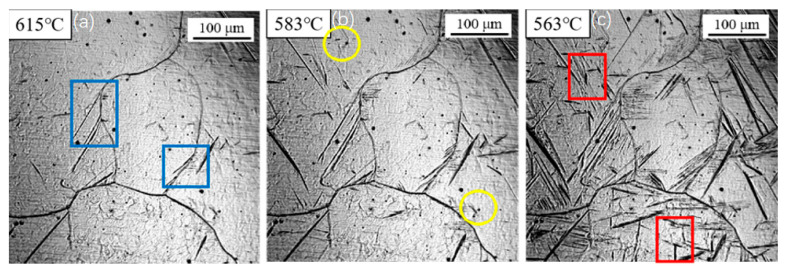
In situ observation of ferrite transformation at 7 °C/s: (**a**) T = 615 °C, growth of sideplate ferrite (SF), region marked by the blue box; (**b**) T = 583 °C, nucleation of acicular ferrite (AF) on an inclusion, region marked by the yellow circle; (**c**) T = 563 °C, formation of secondary ferrite, region marked by the red box.

**Figure 8:**
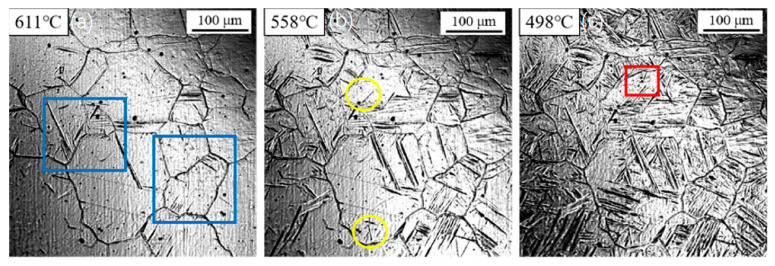
In situ observation of ferrite transformation at 10 °C/s: (**a**) T = 611 °C, growth of sideplate ferrite (SF)region marked by the blue box; (**b**) T = 558 °C, nucleation of acicular ferrite (AF) on an inclusion, region marked by the yellow circle; (**c**) T = 498 °C, formation of secondary ferrite, region marked by the red box.

The effect of cooling rate on the nucleation and growth of acicular ferrite was quantified by measuring the average number of effective nucleation sites and the transformation start temperature for each phase constituent. The variations in the start and finish temperatures of the acicular ferrite (AF) and sideplate ferrite (SF) transformations under different cooling rates were determined via in situ observation, with the results presented in Figure 4, Figure 5, Figure 6, Figure 7 and Figure 8.

Figure 4 presents the transformation sequence observed during cooling at 1 °C/s. The transformation start temperatures for sideplate ferrite (SF), intragranular primary ferrite, and intragranular secondary ferrite were determined to be 668 °C, 647 °C, and 620 °C, respectively. As the temperature decreased, the number, length, and width of the SF gradually increased. Simultaneously, acicular ferrite (AF) nucleated on inclusions within the austenite grains. Finally, secondary ferrite was induced to form on the broad faces of the pre-existing ferrite plates. It is noteworthy that some ferrite laths were observed to form in the grain interiors without an apparent association with inclusions. This can be attributed to the two-dimensional nature of the in situ observation technique; these laths are likely either nucleated on inclusions, located beneath or above the observed plane, or are secondary ferrite plates induced by other subsurface ferrite structures.

Figure 5 displays the phase transformation sequence observed under a cooling rate of 3 °C/s. The transformation start temperatures for sideplate ferrite (SF), intragranular primary ferrite, and intragranular secondary ferrite were measured at 629 °C, 590 °C, and 577 °C, respectively. Notably, all three transformation temperatures exhibited a decrease compared to those observed at the slower cooling rate of 1 °C/s (Figure 4).

Figure 6 illustrates the microstructural evolution during continuous cooling at a rate of 5 °C/s. The transformation start temperatures for sideplate ferrite (SF), intragranular primary ferrite, and intragranular secondary ferrite remained relatively consistent, recorded at approximately 630 °C, 588 °C, and 564 °C, respectively. In contrast to the slower cooling rates previously examined, a significant surge in ferrite nucleation density was observed. Nucleation occurred nearly simultaneously at numerous sites throughout the austenite grain interiors, exhibiting an explosive-like increase in nucleation events.

Figure 7 presents the transformation behavior under a cooling rate of 7 °C/s. Compared to the transformation at 5 °C/s, a further decrease in the transformation start temperatures was observed. Sideplate ferrite (SF) formed at 615 °C, followed by intragranular primary ferrite at 583 °C and intragranular secondary ferrite at 563 °C. As the cooling rate increased, the growth rate of the sideplate ferrite increased significantly, with its plates extending across almost the entire austenite grain.

Figure 8 shows the in situ observation results of ferrite transformation during cooling at 10 °C/s. The transformation temperatures for sideplate ferrite (SF), intragranular primary ferrite, and intragranular secondary ferrite were measured at 611 °C, 558 °C, and 498 °C, respectively. Due to the decreased transformation temperature, the prior austenite grains were partitioned into fine micro-zones. Consequently, induced nucleation of secondary ferrite was scarcely observed under these conditions.

The transformation temperatures of various microstructural constituents were statistically summarized in Table 2. As the cooling rate increased, the transformation start temperatures of both sideplate ferrite (SF) and acicular ferrite (AF) decreased. Specifically, when the cooling rate increased from 1 °C/s to 10 °C/s, the transformation start temperature of SF decreased from 668 °C to 611 °C, while that of AF decreased from 647 °C to 558 °C. The transformation temperature of intragranular secondary ferrite also exhibited a decline from 620 °C to 498 °C.

Notably, within the cooling rate range of 3–5 °C/s, the transformation temperatures remained relatively stable, during which a sharp increase in AF formation was observed. This phenomenon indicates that the potential for intragranular acicular ferrite formation is significantly enhanced within this intermediate cooling range. However, when the cooling rate exceeds this optimal range, less desirable lath-shaped microstructures, which can be detrimental to mechanical properties, tend to form within the grains.

**Figure 9:**
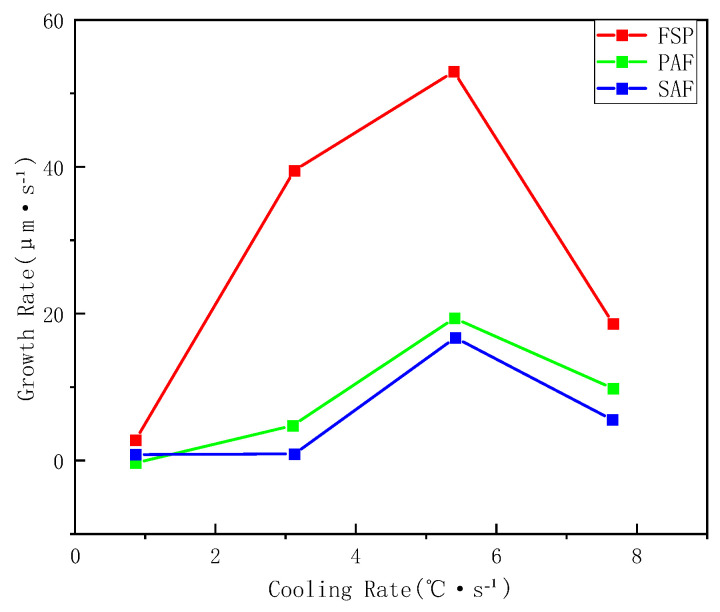
Growth rates of ferrite under different cooling conditions.

Utilizing the high-temperature confocal microscope’s capability to record the phase transformation at 3 frames per second, the growth rates of various ferrite types were determined by measuring the lengthening of ferrite laths. Figure 9 presents the growth rates of sideplate ferrite (SF), primary ferrite, and secondary ferrite under different cooling conditions. The growth rates of all three types initially increase and subsequently decrease with increasing cooling rate, peaking at 5 °C/s. This is similar to the conclusion reached by Zou Leilei et al. [36] on the phase transformation behavior of non-quenched-and-tempered steel.

As sideplate ferrite belongs to the Widmanstätten ferrite family and exhibits a preferential growth direction, its growth rate is significantly higher than that of both primary and secondary ferrite. The growth rates of the latter two are comparable, though the growth rate of the secondary ferrite is slightly lower than that of the primary ferrite. This difference can be attributed to the lower transformation start temperature of secondary ferrite, where the slower diffusion rate at reduced temperatures limits its growth kinetics. This observation further confirms that the secondary ferrite is essentially a product of ferrite transformation occurring at relatively lower temperatures. Its growth is diffusion-controlled and is consequently strongly influenced by carbon diffusion.

The formation of new phases is influenced by factors such as undercooling and carbon diffusion. Carbon atoms diffuse from the ferrite into the surrounding austenite, leading to carbon enrichment in the austenite, which subsequently alters the growth rate of later-forming ferrite. In the initial stage, the rate of new phase formation is relatively slow. As the cooling rate increases, a greater driving force for phase transformation is generated, thereby accelerating the transformation process. However, when the cooling rate exceeds 6 °C/s, the temperature range for phase transformation narrows, and the rate of carbon diffusion decreases. This results in slower transformation kinetics and a reduced ferrite formation rate. Under the combined effects of undercooling and elemental diffusion, the growth rate of ferrite first increases and then decreases with increasing cooling rate. Consistent with the viewpoint proposed by Xiong et al. [37], on the isothermal decomposition behavior of microalloyed steel, in situ observations further provide direct evidence for its kinetic process.

### 3.3. Evolution of Microstructure

**Figure 10:**
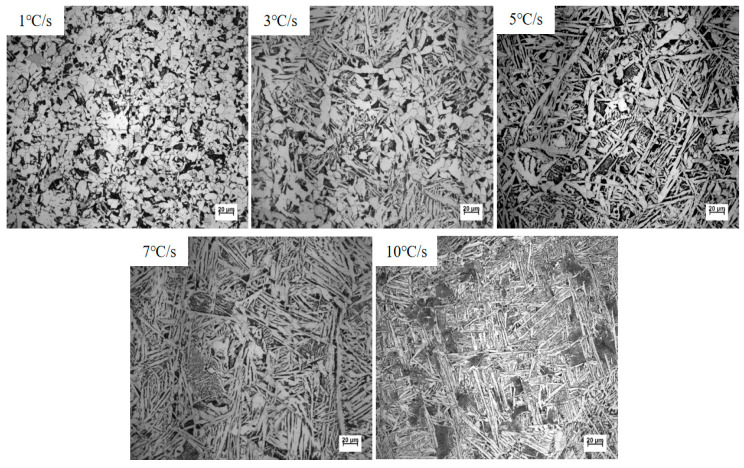
Room-temperature microstructures under different cooling rates.

The metallographic microstructure of samples cooled to room temperature at various rates was observed after the standard preparation procedures involving grinding, polishing, and etching. As shown in Figure 10, the resulting microstructures primarily consist of polygonal ferrite (PF), sideplate ferrite (SF), and acicular ferrite (AF).

A detailed analysis of the metallographic structures in Figure 10 is presented as follows:

At a cooling rate of 1 °C/s, a considerable amount of polygonal ferrite (PF) formed, with no coarse sideplate ferrite (SF) observed. Meanwhile, a small amount of intragranular ferrite (IGF) was nucleated by inclusions within the austenite grains.

When the cooling rate increased to 3 °C/s, the fraction of polygonal ferrite decreased compared to that at 1 °C/s, while the content of intragranular acicular ferrite (AF) increased. Sideplate ferrite remained absent.

At a cooling rate of 5 °C/s, a small amount of sideplate ferrite was formed. In addition, the length of the intragranular acicular ferrite laths increased.

Under the higher cooling rates of 7 °C/s and 10 °C/s, grain boundary ferrite (GBF) first formed along the prior austenite grain boundaries, followed by extensive Widmanstätten structures and sideplate ferrite. The growth space for intragranular ferrite was significantly constrained, leading to a rapid decrease in the volume fraction of inclusion-induced intragranular ferrite.

The volume fractions of intragranular primary ferrite and secondary ferrite were statistically evaluated using the systematic manual point-count method according to the ASTM E562-19 standard. To ensure high statistical accuracy and computational efficiency, a grid of 100 points was employed with a relative accuracy of 10%. Twenty different fields were selected for each sample. The area fractions of the microstructural constituents under different cooling rates are summarized in Table 3.

As the cooling rate increased from 1 °C/s to 10 °C/s, the area fraction of acicular ferrite (AF) exhibited an initial increase followed by a decrease, rising from 26% to a maximum of 74% at 5 °C/s, and then declining to 25%. These results indicate that to obtain a high proportion of acicular ferrite in the experimental steel, the cooling rate should be controlled within the range of 3–7 °C/s. Excessively high cooling rates, even in the presence of favorable inclusions, significantly inhibit the formation of acicular ferrite.

The aspect ratio of intragranular ferrite laths, measured using the software on the metallographic microscope, revealed that the average value increased progressively as the cooling rate rose from 1 °C/s to 7 °C/s, as summarized in Table 4.

According to the diffusion-controlled growth model (modified Zener-Hillert equation), it can be inferred that.

(1) $$\lambda =\frac{v_{tip}}{v_{lat}}$$

(2) $$v_{tip}=\frac{D_{C}∆T}{XT_{e}}$$

(3) $$v_{lat}=\frac{\gamma \delta }{\eta W}$$

where

*Dc*: Diffusion coefficient of carbon in austenite;

Δ*T*: Degree of undercooling;

*X*: Diffusion distance, representing the length over which carbon atoms diffuse from ferrite into austenite, which is related to the transformation time;

*Te:* Equilibrium transformation temperature;

*γ:* Interfacial energy;

*δ*: Section thickness;

*η*: Interface migration resistance coefficient;

*W*: Width of the acicular ferrite lath.

The aspect ratio, λ, is defined as the ratio of the longitudinal growth rate (*Vtip*) to the lateral thickening rate (*Vlat*) of acicular ferrite. Te denotes the thermodynamic equilibrium temperature between austenite and ferrite. A higher cooling rate results in a greater degree of undercooling (Δ*T*), a shorter transformation time, and a reduced diffusion distance (*X*). These conditions promote a higher longitudinal growth rate of acicular ferrite, thereby leading to a larger aspect ratio. The calculation results are consistent with experimental observations: higher cooling rates induce greater undercooling during ferrite transformation, which facilitates preferential growth of ferrite laths along the longitudinal direction [38].

While the model described above is primarily based on diffusion-controlled growth, it is important to note that the growth of acicular ferrite also involves shear transformation as the temperature decreases. Therefore, for more accurate parameter calibration, it is necessary to incorporate additional factors such as interface migration rate and dislocation slip parameters into the model for further refinement.

### 3.4. Microhardness Evaluation and Discussion

**Figure 11:**
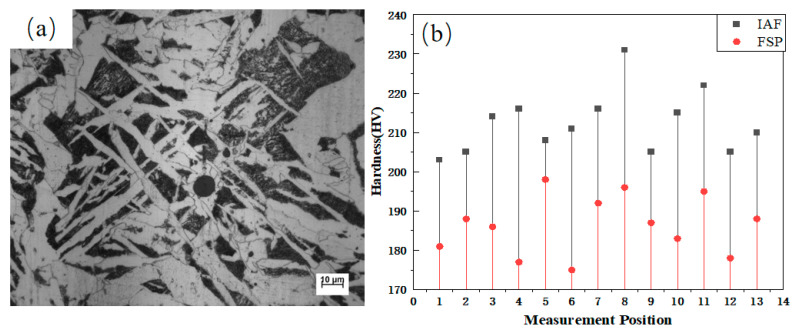
Morphology and mechanical properties of intragranular ferrite interlocking structure: (**a**) microstructure; (**b**) microhardness distribution.

Owing to the constraints imposed by the sample dimensions, the evaluation of the mechanical response of the microstructure to different cooling processes in this section is mainly based on microhardness measurements. Figure 11a displays a typical microstructure of the sample cooled at 5 °C/s. Grain boundary ferrite (GBF) formed along the austenite grain boundaries, while radiated acicular ferrite (AF) nucleated on inclusions within the grains. Microhardness measurements were taken at 13 locations within intragranular AF interlocking regions and 13 locations in non-interlocking regions on this sample, and the average values are presented in Figure 11b.

The microhardness (HV_0_._1_) of the AF interlocking zones reached 212.5, which is approximately 14% higher than that of the other regions (186.5). This result clearly indicates that the hardness of the interlocking microstructure is superior to that of non-interlocking areas within the same sample, demonstrating that acicular ferrite effectively enhances the strength of the steel.

## 4. Discussion

### 4.1. Inclusion Characteristics and Their Influence on Acicular Ferrite Nucleation

**Figure 12:**
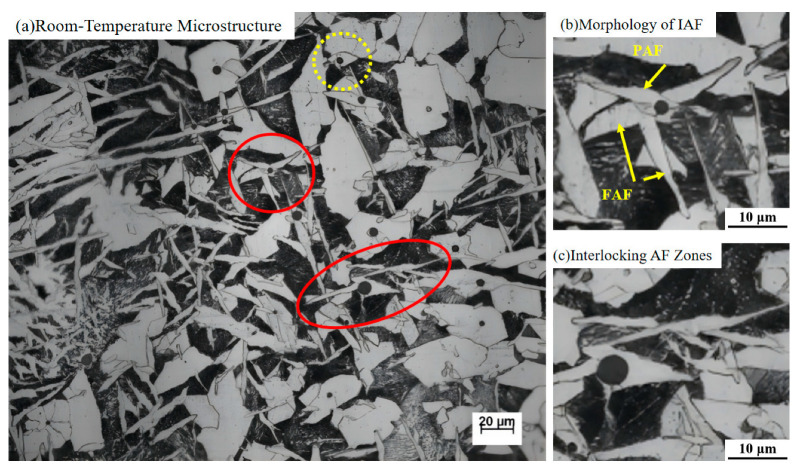
Room-temperature microstructural morphology of the experimental steel. (**a**)Room-Temperature Microstructure, (**b**)Morphology of IAF, (**c**)Interlocking AF Zone. The red and yellow circles mark the inclusion-induced interlocking ferrite structure and blocky ferrite structure, respectively. The yellow arrow indicates the nucleation sites of both primary and secondary acicular ferrite.

A typical metallographic microstructure of the sample cooled at 5 °C/s is shown in Figure 12, which reveals a considerable amount of intragranular ferrite interlocking structure, as outlined by the solid red circle in Figure 12a. The microstructure features multiple instances of inclusion-induced primary acicular ferrite (AF) and subsequently induced secondary AF, each exhibiting distinct morphologies and orientations. Most primary AF laths radiate from inclusions, while the secondary AF nucleates and grows on the broad faces of the primary AF, often with significantly different growth directions, as illustrated in Figure 12b. The AF laths initiated by inclusions undergo hard impingement with adjacent ferrite laths, ultimately forming an interlocked and irregular microstructure, as indicated by the arrow in Figure 12b. In addition, some irregular blocky primary ferrite grains nucleate on inclusions and grow simultaneously in multiple directions, eventually encapsulating the inclusions, as indicated by the dashed circle in Figure 12a.

**Figure 13:**
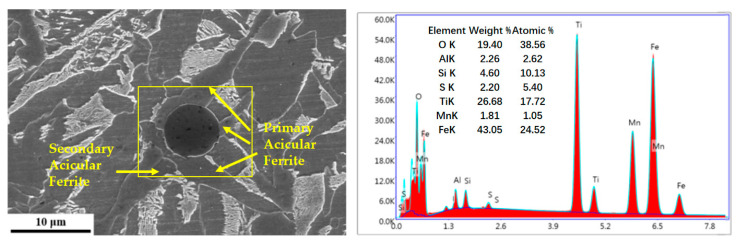
Morphology and composition of inclusion. (the fangk rrow indicates the nucleation sites of both primary and secondary acicular ferrite. EDS analysis point (boxed area)).

Morphological and compositional analysis of the inclusion that induced the nucleation of intragranular primary ferrite was conducted using scanning electron microscopy (SEM) coupled with energy-dispersive X-ray spectroscopy (EDS), as shown in Figure 13. This inclusion acted as an effective nucleation site for acicular ferrite (AF), with several ferrite laths growing radially outward from it. Furthermore, new AF laths formed on the sides of the inclusion-induced AF, indicating a phenomenon of induced nucleation.

Both the primary AF nucleated by the inclusion and the secondary AF, resulting from induced nucleation, formed and grew within the prior austenite grains. This nucleation and growth mode substantiates the view established in prior research that inclusions can induce the growth of multiple ferrite laths. [39,40,41] This microstructure effectively partitioned the original austenite grains into isolated smaller regions, thereby refining the grain size.

The effective inclusion was approximately spherical and promoted the radial growth of multiple primary AF laths. The EDS spectrum on the right corresponds to the boxed area in the left-hand SEM image, identifying the composition of the nucleation site. The EDS spectrum showed prominent peaks of O, Al, Si, S, Ti, and Mn, indicating that the effective inclusions in the steel were complex Ti–Al–Mn–O–S compounds.

**Figure 14:**
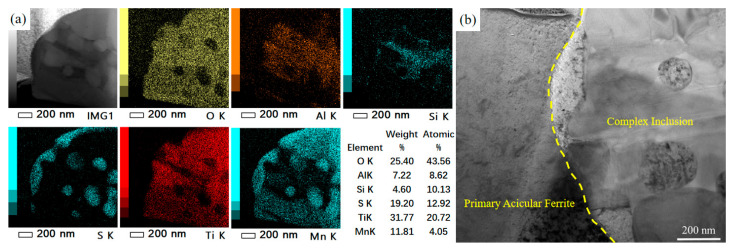
TEM-EDS analysis of the inclusion: (**a**) EDS elemental mapping; (**b**) Interface characterization between MnS and primary ferrite.

Further analysis of the inclusion was performed using transmission electron microscopy (TEM). The morphology and corresponding EDS elemental mapping results are shown in Figure 14. It can be observed that O, Ti, and Mn are uniformly distributed, forming TiOx and MnO. TiOx is present throughout almost the entire inclusion, indicating that it constitutes the matrix. The distributions of S and Mn overlap, confirming the presence of MnS, which aggregates into spherical particles dispersed in the center of the inclusion and also forms an outer MnS band attached to the surface of the inclusion, as shown in Figure 14a.

The close-spaced interface between MnS and the primary ferrite suggests that the primary ferrite nucleates on the outer MnS band of the inclusion. This demonstrates the critical role of MnS in promoting the nucleation of intragranular primary ferrite, as illustrated in Figure 14b. Although Al and Si also exhibit partial overlap in their distributions, the broader distribution of Al suggests the presence of both aluminosilicate (Al_2_O_3_·SiO_2_) and single-phase Al_2_O_3_.

The above analysis indicates that this inclusion evolved from a TiOx-based precursor, ultimately developing into a spherical, multi-phase complex composed of TiOx-Al_2_O_3_·SiO_2_-MnO-MnS. This type of multi-phase inclusion effectively promotes the nucleation of intragranular primary ferrite. This is consistent with the oxide metallurgy theory regarding the ability of multi-phase inclusions to promote nucleation. [42,43]. These findings are consistent with the conclusions reached by Wall et al. [44,45], who reported that acicular ferrite (AF) nucleates on the surface of MnS or at the interface between MnS and oxides. Multi-layered composite inclusions are thus highly conducive to AF nucleation.

### 4.2. Dislocation Structure and Strengthening Mechanisms

**Figure 15:**
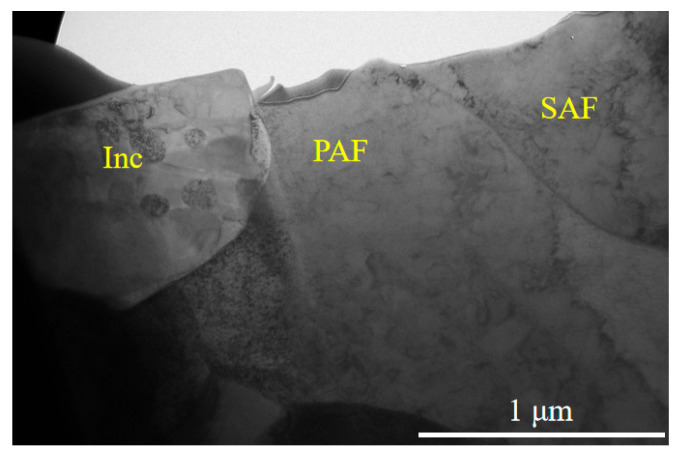
Nucleation of intragranular ferrite on inclusion ((Inc: Inclusion; PAF: Primary Acicular Ferrite; SAF: Secondary Acicular Ferrite).

The TEM micrograph in Figure 15 shows the interfacial morphology between the primary and secondary ferrite. The primary ferrite lath extends and grows in a specific direction within the observed plane, ultimately forming an acicular morphology. The secondary ferrite nucleates at the grain boundary of the primary ferrite and grows along a different direction. The close connection at their interface suggests that the secondary ferrite likely nucleated directly on the boundary of the primary ferrite through induced nucleation, rather than resulting from hard impingement after independent growth.

A high density of dislocations with various configurations—including tangled lines, loops, and clusters—is observed both within the primary ferrite and at its boundaries. The dislocation density is significantly higher in the primary ferrite than in the secondary ferrite. Using the grid method, the measured areas of the primary and secondary ferrite were approximately 1.062 μm^2^ and 0.335 μm^2^, respectively, with approximately 240 and 27 dislocation lines counted in each. According to the standard dislocation density formula.

(4) $$\rho =\frac{nl}{lA}=\frac{n}{A}$$

The calculated dislocation densities are approximately 2 × 10^8^ /mm^2^ for the primary ferrite and 8 × 10^7^ /mm^2^ for the secondary ferrite—differing by an order of magnitude.

This indicates that the high dislocation density near the grain boundary plays a critical role in the induced nucleation of the secondary ferrite. The high density of dislocations within the primary ferrite causes severe lattice distortion and high strain energy, which provides a potent driving force for the induced nucleation of secondary ferrite. Furthermore, solute atoms can segregate along dislocation lines, leading to local enrichment of carbon. This increased carbon saturation enhances the driving force for nucleation. Additionally, dislocation pipes act as short-circuit diffusion paths, facilitating discontinuous diffusion of carbon atoms and reducing the activation energy for diffusion, thereby promoting the growth of the secondary ferrite.

### 4.3. Grain Boundary Characteristics and Toughening Mechanism

**Figure 16:**
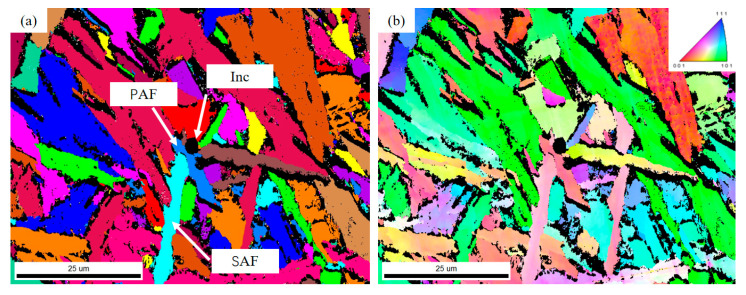
EBSD analysis of intragranular ferrite: (**a**) grain boundary map; (**b**) inverse pole figure (IPF) map showing crystal orientations.

Electron backscatter diffraction (EBSD) was employed to analyze the grain orientation and grain boundary characteristics of the interlocking ferrite microstructure, as shown in Figure 16. Figure 16a presents the grain distribution map, where different colors represent different ferrite grains. Intragranular acicular ferrite (IAF) nucleated by inclusions and subsequently induced secondary IAF can be observed, with extensive interweaving between ferrite grains, forming an interlocked IAF structure. Figure 16b shows the inverse pole figure (IPF) map representing crystal orientations. The grains exhibit random orientations, with four primary AF laths nucleating on the same inclusion and growing in distinct directions, resulting in significant misorientation. The secondary IAF also displays different orientations from the primary IAF, indicating inconsistent crystallographic relationships between both types of acicular ferrite and the prior austenite.

Numerous ferrite grains interweave or undergo hard impingement. Some ferrite grains are segmented by other ferrite laths, yet the segmented portions maintain similar crystallographic orientations. This suggests that these fine ferrite regions originally belonged to the same grain, which underwent subtle orientation adjustments during growth and interlocking with surrounding ferrite, ultimately appearing as separate but similarly oriented grains in the two-dimensional observation plane.

**Figure 17:**
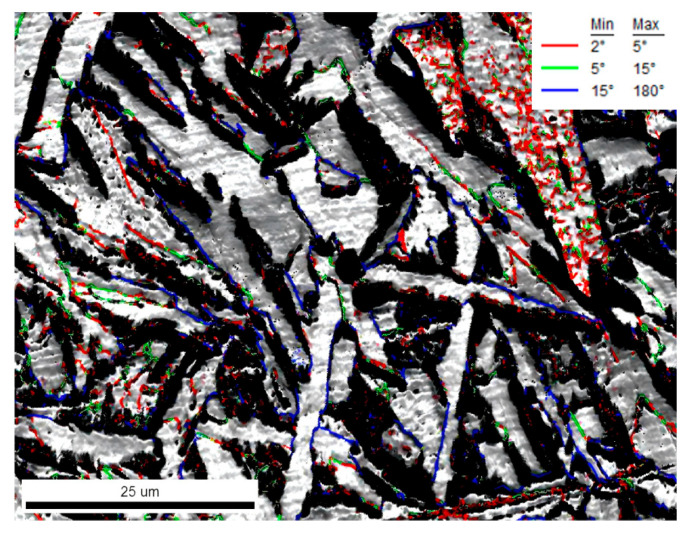
Grain boundary distribution in intragranular ferrite.

The grain boundary configuration between ferrite grains is illustrated in Figure 17, where the red lines represent low-angle grain boundaries (LAGBs) of 2–5°, green lines denote LAGBs of 5–15°, and blue lines indicate high-angle grain boundaries (HAGBs) of 15–180°. The boundaries between the secondary IAF and primary IAF are predominantly LAGBs of 5–15°, while the boundaries between ferrite grains formed by hard impingement are HAGBs, represented by blue lines. The induced nucleation of secondary ferrite and extensive interweaving and hard impingement among ferrite grains collectively contribute to the formation of the interlocked acicular ferrite microstructure.

The EBSD analysis demonstrates that the acicular ferrite (AF) zone exhibits a highly tortuous and interlocked grain boundary network, as revealed by the grain boundary misorientation map. This interlaced high-angle grain boundary structure effectively enhances toughness by forcing crack propagation along meandering paths. Furthermore, the statistically refined grain size in the AF zone (approximately 20 μm), as determined by EBSD analysis, is significantly finer than that in the polygonal ferrite (PF) region, contributing substantially to grain refinement strengthening according to the Hall-Petch relationship. These distinctive structural characteristics provide the microstructural foundation for the simultaneous improvement of both strength and toughness in the material.

## 5. Conclusions

In this study were systematically investigated the effects of cooling rate on the formation of acicular ferrite (AF) and the resultant mechanical properties in EH36 steel through in situ observation via high-temperature laser confocal microscopy and multi-scale characterization. The main conclusions are as follows:

1. The cooling rate significantly influences AF formation. Increasing the cooling rate reduces the AF nucleation temperature (e.g., the primary AF start temperature decreased from 647 °C to 558 °C as the cooling rate increased from 1 °C/s to 10 °C/s). The optimal cooling rate range was identified as 3–5 °C/s. At 5 °C/s, AF exhibited the highest growth rate (approximately 13 μm/s), the highest volume fraction (74%), and the optimal aspect ratio (5.97). Excessively low cooling rates (e.g., 1 °C/s) promote the formation of polygonal ferrite, while excessively high cooling rates (e.g., 10 °C/s) lead to coarse Widmanstätten structures. The balance between undercooling and carbon diffusion is identified as the key governing factor.

2. Complex TiOx-Al_2_O_3_·SiO_2_-MnO-MnS inclusions act as effective nucleation sites for AF. The outer MnS layer plays a critical role in reducing interfacial energy and facilitating the radial growth of AF. This core–shell inclusion structure effectively lowers the nucleation barrier.

3. Secondary AF nucleates on the broad faces of primary AF laths via induced nucleation. The high dislocation density in primary AF (2.5 times higher than in secondary AF) provides a driving force for secondary ferrite nucleation through strain energy and local carbon enrichment. This high-density dislocation structure results in a 14% increase in microhardness compared to sideplate ferrite, confirming that the interlocked AF structure enhances both strength and toughness.

4. The acicular ferrite structure significantly strengthens and toughens the material by dividing prior austenite grains into fine micro-scale regions and introducing a high fraction of high-angle grain boundaries. This unique interlocking microstructure is identified as the key mechanism for the enhanced mechanical properties.

Based on these findings, this study not only provides a theoretical basis for optimizing the thermo-mechanical control process (TMCP) of heavy steel plates, but also offers valuable insights for the design of high-performance steel materials through oxide metallurgy and microstructure control, as well.

## Figures and Tables

**Table 1:** Chemical composition of experimental steel (wt.%).

Element	C	Si	Mn	Al	Ti	Nb	V	Mo	P	S
**wt.%**	**0.18**	**0.19**	**0.85**	**0.007**	**0.048**	**0.02**	**0.04**	**0.09**	**0.018**	**0.006**

**Table 2:** Transformation temperature (°C/s) of intragranular ferrite.

	Cooling Rate/(℃·S^−1^)	1	3	5	7	10
Microstructure						
FSP		668	629	630	615	611
Primary IAF		647	590	588	583	558
Secondary IAF		620	577	564	563	498

**Table 3:** Area fraction of intragranular acicular ferrite (AF) under different cooling rates.

Cooling Rate/(°C·S^−1^)	1	3	5	7	10
Primary Ferrite/vol%	15	34	42	24	16
Secondary Ferrite/vol%	11	28	32	20	9

**Table 4:** Aspect ratio of acicular ferrite under different cooling rates.

Cooling Rate/(°C·s^−1^)	1	3	5	7
Minimum	1.79	1.61	1.97	2.40
Maximum	10.82	15.03	32.31	14.52
Average	4.51	5.66	5.97	6.83

## Data Availability

The original contributions presented in this study are included in the article. Further inquiries can be directed to the corresponding author.
